# Plant-Derived Anticancer Compounds as New Perspectives in Drug Discovery and Alternative Therapy

**DOI:** 10.3390/molecules26041109

**Published:** 2021-02-19

**Authors:** Cristina Adriana Dehelean, Iasmina Marcovici, Codruta Soica, Marius Mioc, Dorina Coricovac, Stela Iurciuc, Octavian Marius Cretu, Iulia Pinzaru

**Affiliations:** 1 Research Center for Pharmaco-Toxicological Evaluations, Faculty of Pharmacy, “Victor Babes” University of Medicine and Pharmacy Timisoara, Eftimie Murgu Square No. 2, RO-300041 Timisoara, Romania; cadehelean@umft.ro (C.A.D.); iasmina.marcovici@umft.ro (I.M.); dorinacoricovac@umft.ro (D.C.); iuliapinzaru@umft.ro (I.P.); 2 Faculty of Pharmacy, “Victor Babeș” University of Medicine and Pharmacy Timisoara, Eftimie Murgu Square No. 2, RO-300041 Timisoara, Romania; 3 “Victor Babeș” University of Medicine and Pharmacy Timisoara, Faculty of Medicine, Eftimie Murgu Square No. 2, RO-300041 Timisoara, Romania; siurciuc@umft.ro (S.I.); tavi@octaviancretu.ro (O.M.C.)

**Keywords:** bioactive compounds, doxorubicin, paclitaxel, vincristine, resveratrol, curcumin, rutin, betulinic acid, antitumoral effect, chemoprevention

## Abstract

Despite the recent advances in the field of chemically synthetized pharmaceutical agents, nature remains the main supplier of bioactive molecules. The research of natural products is a valuable approach for the discovery and development of novel biologically active compounds possessing unique structures and mechanisms of action. Although their use belongs to the traditional treatment regimes, plant-derived compounds still cover a large portion of the current-day pharmaceutical agents. Their medical importance is well recognized in the field of oncology, especially as an alternative to the limitations of conventional chemotherapy (severe side effects and inefficacy due to the occurrence of multi-drug resistance). This review offers a comprehensive perspective of the first blockbuster chemotherapeutic agents of natural origin’s (e.g. taxol, vincristine, doxorubicin) mechanism of action using 3D representation. In addition is portrayed the step-by-step evolution from preclinical to clinical evaluation of the most recently studied natural compounds with potent antitumor activity (e.g. resveratrol, curcumin, betulinic acid, etc.) in terms of anticancer mechanisms of action and the possible indications as chemotherapeutic or chemopreventive agents and sensitizers. Finally, this review describes several efficient platforms for the encapsulation and targeted delivery of natural compounds in cancer treatment

## 1. Introduction

Nature stands as an infinite and inexhaustible resource for the development of novel effective drugs, chemotypes and pharmacophores [1]. Medicinal herbs have a wide outspread globally and, in recent years, have become of essential importance in treating diseases. Their therapeutic role is provided by bioactive molecules, the components of the plant’s chemical core [2]. Since ancient times, natural compounds have played an important role in the treatment and prevention of various pathologies, forming the backbone of the traditional system of healing [3,4]. Furthermore, terrestrial plants (e.g. *Artemisia annua* L., *Camptotheca acuminata* Decne., *Gingko biloba* L., *Curcuma longa* L., *Podophyllum peltatum* L., *Taxus brevifolia*, *Taxus baccata*, *Combretum caffrum*, *Euphorbia peplus*, etc.) still dominate in current-day therapeutic approaches, since the plant-derived compounds comprise a large portion of current pharmaceutical agents, most notably in the areas of antibiotherapy and chemotherapy [4].

The role of natural compounds as drugs or as a basis for the development of new drugs is thoroughly explained in the most recent review published by Newman and Cragg [5]. According to their data, out of a total of 1881 new approved drugs (antibacterial, antifungal, antiviral, antiparasitic, antitumor, etc.) in the last four decades, 929 have natural origin and fall into the class of biological macromolecules, unaltered natural products, botanical drugs, or derivatives of natural products or vaccines, while the other 952 drugs are classified as synthetic drugs, synthetic drugs with natural product pharmacophores or as drugs that mimic a natural product [5].

The use of herbal medicines has been widely embraced as a complementary or alternative option in the oncology field (*Catharanthus roseus, Podophyllum peltatum* L., *Taxus brevifolia* Nutt., *Taxus baccata,* etc.) [3,4]. Therefore, every year, several novel cytotoxic compounds are isolated from plants and constitute new possibilities to fight cancer. Many researchers center their attention on the investigation of naturally occurring molecular entities that might become useful to the pharmaceutical industry [6]. Those who discover compounds exerting antitumor activity during preclinical studies seek out for clinical efficacy confirmation too.

Carcinogenesis, the phenomenon of cancer arising and developing, is a step-by-step process [7] characterized by the progression of distinct molecular changes [8], accumulation of mutations and epigenetic alterations that activate oncogenes, inactivation of tumor suppressor genes, hindrance of DNA repair machinery and disruption of apoptosis mechanisms [7], which compels cells to undergo uncontrolled cellular division [8]. A single alteration is not able to promote tumorigenesis, but a collection of multiple modifications that affect cellular homeostasis is necessary to make cells lose control of proliferation [7]. With each disturbance, cells undergo fundamental changes, leading to tumor initiation, promotion and progression [8]. Tumor initiation is a rapid and irreversible process that begins with the exposure to a carcinogenic agent, followed by its transportation to tissues where it causes DNA mutations. During the promotion stage, which is a long and reversible process, the tumor-initiated cells proliferate, allowing the accumulation of additional mutations. The final stage of neoplastic transformation, progression, occurs after these mutations generate an invasive and metastatic cellular phenotype [8]. Regarding cancer therapy, definitive tumor eradication through surgical resection, radiation therapy, immunotherapy and chemotherapy continues to be part of the current mainstay treatment regimens [4,9]. However, the prevention of carcinogenesis is highly preferable to treatment [4], as cancer is largely a preventable disease that can be attributed to lifestyle factors (such as smoking and nutritional status) [10]. The concept of chemoprevention is gaining more public attention especially because it is cost-effective and avoids the toxic side effects following cancer treatment [10].

The benefits of natural compounds as reduced adverse effects and the capacity to impact on multiple signaling pathways involved in the carcinogenesis process could be considered an explanation for the fact that from 240 antitumor drugs approved in the last 40 years, only 29 are strictly synthetic drugs. Moreover, in the past 10 years, synthetic compounds with pharmacophore of natural origin that mimic the natural product effect were approved as antitumor drugs [5].

Phytochemicals have been in the spotlight of cancer research since its early beginnings, as they were among the first antineoplastic drugs discovered (e.g., leucovorin in 1950, carzinophilin in 1954, vincristine in 1963, actinomycin D in 1964, etc.) [5]. Furthermore, their study continued to the present day. It is of great importance to mention that natural compounds not only serve as chemotherapeutic agents, but also as adjuvants in cancer treatment.

This review offers a new perspective regarding the role of natural compounds in the oncology field by summarizing the three directions of their anticancer activity: (I) chemotherapeutic agents due to their innate antitumor effect; (II) chemopreventive agents; (III) sensitizers in multi-drug resistance.

## 2. Natural Compounds Recognized as Chemotherapeutic Agents

### 2.1. Natural Compounds Approved as Chemotherapeutic Agents 

**Figure 1:**
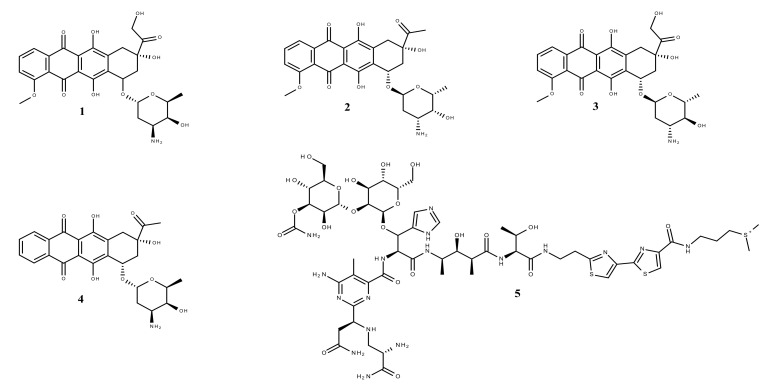
Chemical structures of (1) doxorubicin, (2) daunorubicin, (3) epirubicin, (4) idarubicin and (5) bleomycin.

**Figure 2:**
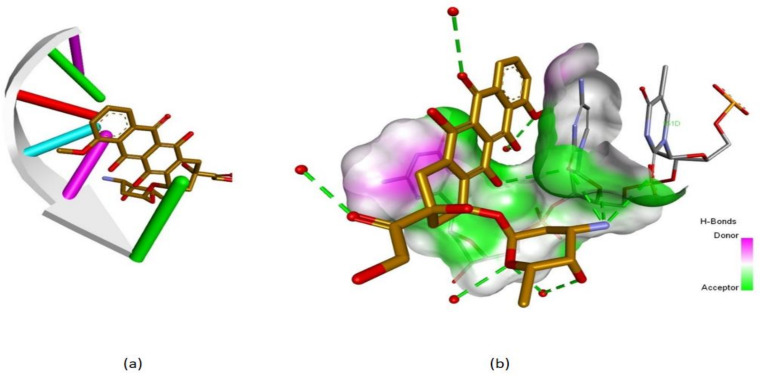
(**a**) Crystallographic 3D structure of doxorubicin (gold) in complex with DNA (PDB ID: 151D), d(CGATCG) and (**b**) the anthracycline structure is strongly bound to DNA base pairs through multiple hydrogen bonds (HBs) depicted as green dotted lines; DNA-ligand representation was achieved using Biovia Discovery Studio 4.1 (Dassault Systems).

Terrestrial plants, microbes, slime molds and the marine environment represent important sources of novel pharmacologically active molecules, providing a library of promising therapeutic agents [4]. Many blockbuster drugs are directly or indirectly derived from natural products and were or still are in clinical use [1,11,12]. Despite the increased popularity of the synthetic products which led to many drugs existing nowadays [1], the secondary metabolites from plants remain vital for drug design [2], as their core structures serve as templates for the synthesis or semi-synthesis of novel substances for the treatment of diseases affecting humankind [1]. Additionally, while the safety–efficacy report of synthetic drugs remains debatable, the naturally occurring molecules still play a dominant role in the treatment of human ailments [1,4,13] due to their high patient tolerance and acceptance [1]. Conventional chemotherapy drugs (e.g., methotrexate, cisplatin) are associated with severe adverse reactions such as hair loss, gastrointestinal lesion, bone marrow sup-pression, neurologic dysfunction, and drug resistance, while phytocompounds proved to be less toxic and more effective [2,14,15]. Presently, the molecules that occur in nature comprise a large portion of the current-day pharmaceutical agents, most notably in the area of antibiotics and cancer drugs [4], among which 60 to 80% are derived from natural products [16]. Moreover, about one third of the world’s best-selling drugs are natural compounds or their derivatives [16]. The most representative example is the legendary discovery of the first antibiotic penicillin from the fungus *Penicillium notatum*, by Alexander Fleming, who noticed that “around a large colony of a contaminating mold the staphylococcus colonies became transparent and were obviously undergoing lysis” [2,17]. Antibiotics stood out even in the field of cancer treatment, being among the most important of the chemotherapeutic agents. These include members of the actinomycin, ansamycin, anthracycline, bleomycin, epothilone and staurosporine classes [4]. Anthracycline chemotherapy regimens play a central role in the treatment of many cancer types including breast cancer and lymphoma. Doxorubicin (derived from microbes), its precursor daunorubicin, epirubicin and idarubicin (Figure 1) are the most common four anthracyclines, among which the first two are the earliest anthracyclines to be put into clinical practice [18]. Their cytotoxic effects are a result of several mechanistic approaches, including topoisomerase II, DNA and RNA inhibitions. Topoisomerase II decreases DNA supercoiling by cutting DNA double strands during replication. Anthracyclines bind DNA and topoisomerase II isoenzymes, forming a ternary complex that causes double-stranded DNA breaks. Topoisomerase IIα (TopIIα) is the most prevalent isoenzyme, being highly expressed in malignant cells [18]. When bound to TopIIα, anthracyclines block its catalytic activity, stabilize DNA breaks and inhibit DNA replication [18]. Moreover, anthracyclines intercalate with DNA bases by forming adducts and inhibit the activity of DNA and RNA polymerases that result in the blockage of DNA and RNA synthesis. The interactions of anthracyclines with nucleic acids, especially DNA, are very well documented starting from the 1990s. The extensive research conducted allowed the observation of binding patterns and base pair interaction behavior between DNA strands and anthracyclines by obtaining 3D crystallographic structures of DNA-anthracycline complexes [19]. These structures show how anthracyclines are strongly bound to DNA base pairs through multiple hydrogen bonds (Figure 2) and outline the mobility changes in base pairs when interacting with these molecules. Nevertheless, despite the great results recorded in tumor regression, anthracycline therapy may result in a high cardiac risk [20]. Bleomycin (Figure 1) belongs to the family of glycopeptide antibiotics and is primarily indicated as an antineoplastic agent. Its mechanism of action involves the oxidative damage of DNA via metal ions binding, which results in the formation of metallobleomycin complexes. Moreover, chromosomal aberrations, chromatid breaks and translocations were observed after the exposure to this antibiotic. Bleomycin received the FDA-approval for the treatment of various malignancies such as squamous cell carcinomas, testicular cancers, Hodgkin and non-Hodgkin lymphomas [21].

**Figure 3:**
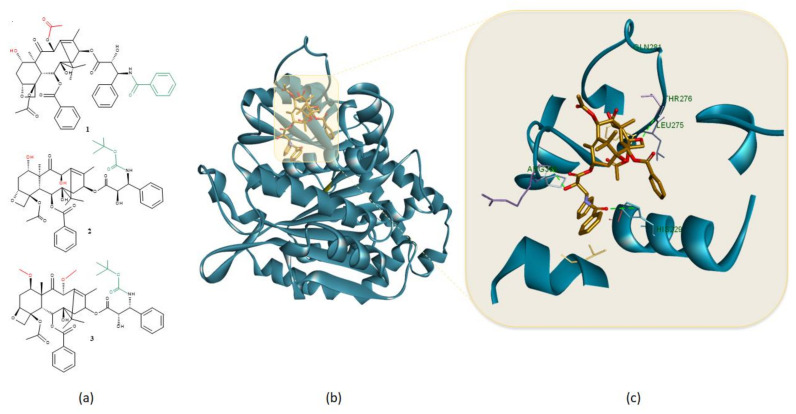
(**a**) Chemical structures of (1) paclitaxel, (2) docetaxel, and (3) cabazitaxel, major structural differences are highlighted in green while alkylation/acylation of OH groups is highlighted in red; (**b**) 3D structure of stabilized microtubule chain A (blue) (PDB ID: 5SYF) in complex with taxol (gold) and (**c**) important HBs (green dotted lines) formed by the taxane structure within the β-tubulin binding site. Protein-ligand representation was achieved using Biovia Discovery Studio 4.1 (Dassault Systems).

*Taxus* species are a veritable source of taxanes which became one of the most important classes of chemotherapeutic drugs in the modern clinical use [4]. However, they have a long history of use in folk medicine against breast and ovarian cancers [20]. There are two relevant drugs belonging to this class of natural anticancer agents—paclitaxel, originally isolated from the bark of *Taxus brevifolia,* and docetaxel, the semisynthetic analogue of a compound abundant in the leaves of *Taxus baccata* (Figure 3a). Even though compounds such as docetaxel and cabazitaxel are taxoid semisynthetic derivatives, important structural modulations occur at the NH group as compared to paclitaxel. Other structural differences are related to the degree of methylation or acetylation of the outer ring OH groups. Microtubules are α,β-tubulin containing components that assemble longitudinally into protofilaments also playing a key role in cell division [22]. The antitumoral effect of taxanes is based on their ability to promote the polymerization of tubulin heterodimers to stabilize microtubules (Figure 3b) and suppress the dynamic changes in microtubules, leading to mitotic arrest. Taxanes stabilize microtubules by binding to the β-tubulin subunit. The strong microtubule stabilization effect relies upon the multiple HB that the taxane compound forms within the β-tubulin binding site (Figure 3c) [22]. Therefore, the derivatization of OH groups in obtaining semisynthetic derivatives is limited to alkylation/acylation, because of the important role that these functional groups play in HB formation. Currently, both paclitaxel and docetaxel are highly recommended in the treatment of breast and non-small cell lung malignancies. Additionally, paclitaxel has also shown efficacy against Kaposi’s sarcoma and ovarian cancer [4]. Their high value in chemotherapy, as well as clinical limitations including solubility issues and toxicity, led to the development of taxoid-analogues which are currently in different stages of clinical trials [4]. For instance, cabazitaxel, which conserves the same mechanism of action [20], proved to be active against several types of malignant neoplasms, including prostate, bladder, brain, ovarian, stomach and urinary tract cancers [4].

**Figure 4:**
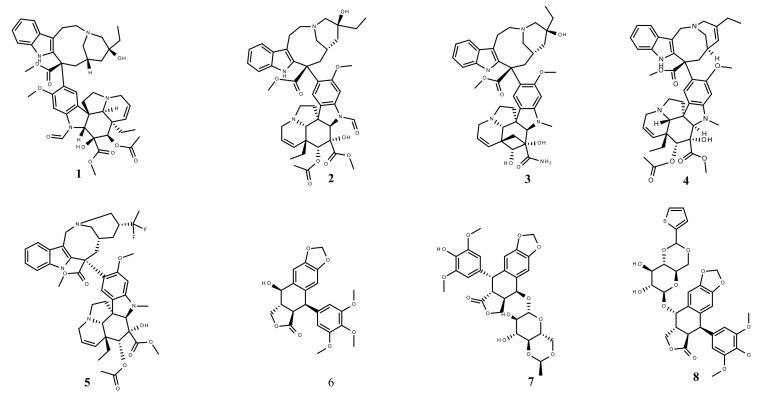
Chemical structures of (1) vincristine, (2) vinblastine, (3) vindesine, (4) vinorelbine, (5) vinflunine, (6) podophyllotoxin, (7) etoposide and (8) teniposide.

**Figure 5:**
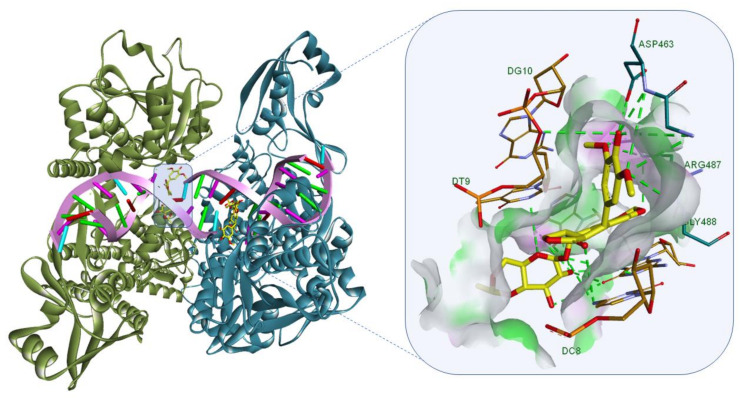
DNA-topoisomerase IIα-etoposide complex (PDB ID: 5GWK); HB (green dotted lines) formed by etoposide (yellow sticks) with interacting amino acids (blue sticks) and nucleotides (orange sticks).

The Vinca alkaloids, vincristine and vinblastine (Figure 4), are the first plant-derived anticancer drugs to enter the clinical use [23]. Originally isolated from the Madagascar periwinkle, *Catharanthus roseus*, they show great antiproliferative activity by disrupting microtubules, causing cell arrest in the metaphase and leading to apoptotic cellular death [4]. They were used as templates for the development of effective semisynthetic derivatives, including vindesine, vinorelbine and its bifluorinated analogue, vinflunine (Figure 4) [4]. Podophyllotoxin, the active constituent of *Podophyllum peltatum* L. which is carrying a long tradition of use in the treatment of skin cancer and warts, contributed to the semisynthesis of two clinically effective derivatives—etoposide and teniposide (Figure 4). Their mechanism of action, in contradiction to podophyllotoxin, which reversibly binds to tubulin [4], consists of the inhibition of the DNA synthesis, breakdown of the DNA stand and stabilization of the topoisomerase II cleavable complex, affecting the late S-G2 interface stage of cell cycle [20]. Crystallographic data show that etoposide, for instance, binds at the interface of DNA and each topoisomerase II chain where HB interactions are formed with both base pairs and amino acid residues (Figure 5). These patterns allowed researchers to design platinum based etoposide derivatives with higher binding capacity and increased selectivity [24].

### 2.2. Natural Compounds with Anticancer Potential

There is a plethora of active agents under ongoing preclinical and clinical trials.

It is important to highlight the significant achievements that were made in this area of research, some secondary plant metabolites being already in clinical use while others are currently tested in clinical trials as anticancer agents [6].

**Figure 6:**
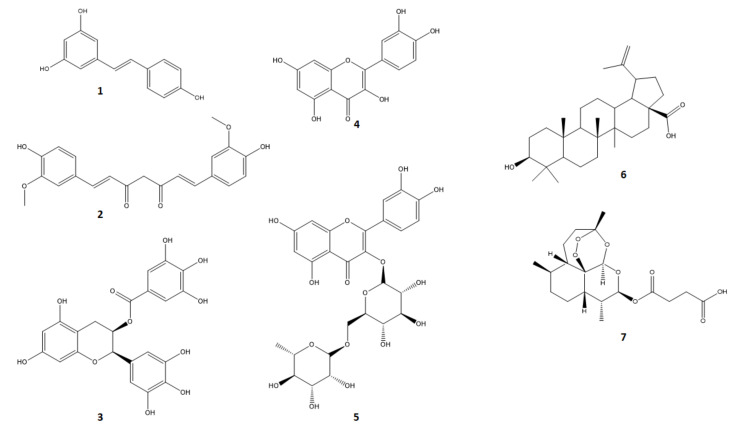
Chemical structures of (1) resveratrol, (2) curcumin, (3) EGCG, (4) quercetin, (5) rutin, (6) betulinic acid and (7) artesunate.

One representative compound intensively studied for its antitumor effect is resveratrol (Figure 6), a phytoalexin and stilbenoid produced by several plants such as grapes, peanuts, cranberries and blueberries [7]. Significant amounts of resveratrol are present in red wine as well [8]. There were several previously described mechanisms for the antitumor activity of resveratrol, such as: (i) an anti-inflammatory effect by decreasing the expression of transcription factors (NF-κB) and mediators associated with inflammation (prostaglandin E2) [7]; (ii) a proapoptotic effect by interfering with multiple signaling pathways [8], and (iii) an antioxidant effect manifested in vivo via gene regulation, partially mediated by histone/protein deacetylase sirtuin 1 or nuclear factor-erythroid 2, reduction of generation of mitochondrial superoxide, prevention of the production of superoxide from uncoupled endothelial nitric oxide synthase, and increasing the expression of various antioxidant enzymes [25,26].

Regarding its biological effects, resveratrol exerted a small reduction in cell proliferation in colorectal tissue after ingestion, which is consistent with its antiproliferative activity that has been observed both in vitro (Table 1), on colorectal cells and in vivo (Table 2), on colorectal tissue in rats. Although resveratrol has shown some efficacy in cancer patients, poor bioavailability limits its use. The study conducted by Patel and colleagues investigated the pharmacological activity of resveratrol and its metabolites in twenty colorectal cancer patients who received the compound formulated as uncoated, immediate release caplets, prior to surgical resection, at either 0.5 or 1.0 g daily, for 8 days. They concluded that resveratrol merits further clinical evaluation in order to become an agent used in chemoprevention [27]. However, a clinical study reported that the process of micronization can enhance the absorption of resveratrol across the gastrointestinal tract, increasing its plasma levels after ingestion [28]. The efficacy, safety and pharmacokinetics of resveratrol have been extensively documented in several clinical trials (Table 3).

**Figure 7:**
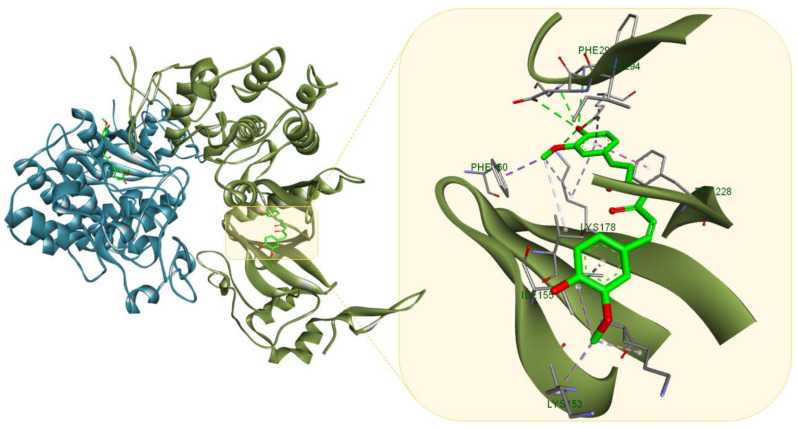
Curcumin (light green) in in complex with DYRK2 (PDB ID: 5ZTN); the compound interacts with the surrounding aminoacid residues through multiple HBs (green dotted lines) and several hydrophobic interactions (purple dotted lines). Protein-ligand representation was achieved using Biovia Discovery Studio 4.1 (Dassault Systems).

**Curcumin** is a diarylheptanoid (Figure 6), belonging to the group of curcuminoids, which are the main components of turmeric (*Curcuma longa*). It has been traditionally used as an anti-inflammatory compound due to its ability to inhibit the activity of NF-κB and COX-2, while suppressing the production of prostaglandins [29]. The antitumoral activities of curcumin are mediated through inhibition of multiple signaling pathways involved in regulation of proliferation, apoptosis, survival, angiogenesis and metastasis, as presented in Table 1 and Table 2. In the ongoing search for possible targets of curcumin that can relate to its antitumoral properties, a recent study reported that curcumin targets dual-specificity tyrosine-regulated kinase 2 (DYRK2), a positive regulator of the 26S proteasome [30]. Curcumin targets DYRK2 with a high specificity by binding to the ATP-binding domain (Figure 7). Researchers also showed that in a breast cancer xenograft model (Table 2), treatment with curcumin significantly reduced tumor burden in immunocompromised mice, exhibiting a similar antitumor effect as compared to clustered regularly interspaced short palindromic repeat (CRISPR)/Cas9-mediated DYRK2 [30].

For instance, curcumin is one of the most extensively studied compounds to date [31]. The anticarcinogenic activity of turmeric has been shown during preclinical studies in various tumor cell lines and xenograft models, leading to phase I and II clinical trials (Table 3). According to these studies, some results are promising and demonstrated the safety of curcumin even at high doses administered over several months [32] with a maximum tolerated dose of 8000 mg/day [31]. However, higher doses of 12,000 mg/day showed minimum toxic effects [31]. Despite its notable therapeutic potential and safety, several studies reported that oral administration of curcumin leads to a low bioavailability due to several causes—poor intestinal absorption, high rate of metabolism and rapid systemic elimination. Among all the pharmacokinetic studies in humans to date, the highest plasma concentration level was reached after the intake of 12 g of curcumin. Interestingly, the low bioavailability of curcumin does not seem to have an impact on its therapeutic effect, according to several clinical studies that have shown a remarkable efficacy despite this limitation [31].

Epigallocatechin-3-gallate (EGCG) (Figure 6) accounts for more than 50% of the total polyphenols found in green tea [33] (*Camelia sinensis* L.) and appears to be the most effective and best-studied constituent that actually entered the phase I and phase II clinical trials for the treatment of different types of cancer including pancreatic, bladder and lung carcinomas [32,34]. However, most of the available epidemiological evidence on tea consumption and cancer prevention in humans has not yielded conclusive results. One of the influencing factors, besides the quantity and quality of the green tea that is consumed, is the low plasma concentrations reached by EGCG [32]. The oral bioavailability of tea polyphenols in humans is low but if the administration is repeated, it leads to a 60% increase in the systemic availability of free EGCG [35]. The safety and limited side effects of EGCG were recorded during clinical trials as well [32].

Quercetin, a major representative of the flavonoid subclass of flavonols, is abundantly present in fruits and vegetables [36]. Quercetin and its glycosides were reported to be active against cancer cells by inducing DNA damage via reactive oxygen species (ROS) (e.g., hydrogen peroxide) generation [9], eliciting a high potential in oncology [36]. The anticancer effect of quercetin is dose-dependent. At low concentrations, this phytocompound has chemopreventive properties due to its antioxidant activity, while at high concentrations it acts as a pro-oxidant, manifesting a chemotherapeutic effect [36]. Several studies revealed the molecular mechanisms of the anticancer effects of quercetin, as presented in Table 1 and Table 2. Quercetin is able to induce cell cycle arrest [37], apoptosis, and necrosis [38] in cancer cells, as well as autophagy, tumor angiogenesis, and metastasis inhibition [39]. In the study conducted by Sturza et al, quercetin caused a dose-dependent inhibitory effect on cellular bioenergetics of murine melanoma cells, modulating glycolytic and mitochondrial pathways for ATP production, which reveals an effective approach in eradicating cancer cells [40].

Rutin, also known as rutoside or sophorin, is a glycoside consisting of quercetin and the disaccharide rutinose. The contribution that rutin might have in cancer prevention and treatment derives from its ubiquitous pharmacological properties. It has antioxidant, anti-inflammatory, antiangiogenic, pro-apoptotic, and antiproliferative activities [41]. This biomolecule was reported to decrease adhesion and migration of human cancerous cells as well [42]. A small part of the in vitro studies that focused on rutin activity, which had continuity in vivo and subsequently reached clinical trials are presented in Table 1, Table 2 and Table 3.

Betulinic acid (Figure 6) is a lupane-type pentacyclic triterpene that extensively spread throughout the plant kingdom. The recent studies that proved the activity betulinic acid possesses against cancer cells [43,44], especially melanoma [45], led to its selection by the National Cancer Institute for addition into the Rapid Access to Intervention in Development (RAID) program. Betulinic acid exhibits significant effects both in vitro (Table 1) and in vivo (Table 2) by manifesting cytotoxicity on various cancer cell lines and suppressing the growth of tumors. There are multiple mechanisms involved in its anticancer activity, the best characterized mechanism being the induction of apoptosis by direct regulation of the mitochondrial apoptotic pathway [6,46]. However, its low water solubility is a drawback in regard of intestinal absorption and, consequently, its bioavailability. Despite its preclinical effectiveness in treating cancer, according to US National Library of Medicine – ClinicalTrials.gov there is only one registered clinical phase I and II study of betulinic acid. Unfortunately, the trial that aimed to evaluate the action of a topical 20% betulinic acid ointment against dysplastic nevi with moderate to severe dysplasia, has been suspended due to funding issues [6]. However, much more effort should be made in order to bring this compound to clinical trials as its potential of becoming an antitumoral drug has been proven via numerous preclinical experiments. Moreover, there are other anticancer molecules currently studied in clinical trials alone or in combination with other anticancer drugs [47].

Artemisinin, a sesquiterpene lactone derived from *Artemisia annua*, is commonly known for its antimalarial activity. Although the artemisinin-mediated cytotoxicity in cancer revolves around the induction of cell growth arrest on human melanoma cells [48], induction of caspase-dependent apoptosis and interference with cancer cell metabolism by reducing its uptake and suppressing ATP and lactate production [49], cell autophagy has been mentioned in a few studies as well [50,51]. Recently, it has been discovered that artemisinin is able to induce a form of iron-dependent, nonapoptotic cell death called ferroptosis [49]. However, not only artemisinin but also its semisynthetic derivatives (e.g. artesunate) possess significant anticancer properties on a variety of cancer types [49]. Artesunate was developed in the 1970s in attempts to treat malaria and since 2019 it is recommended as a component of the first-line treatment of severe malaria in both children and adults. Afterwards, artesunate was proved to have different pharmacological properties, such as: antimetastatic, antiproliferative, proapoptotic and antiangiogenic, becoming one of the compounds intensively researched in the field of oncology today [52].

Ginseng is the name of a group of botanicals in the genus Panax (Araliaceae), which includes species exerting a variety of pharmacological actions [53]. It is claimed to be effective in the treatment of many health problems [54] due to its richness in ginsenosides which are the major bioactive constituents of ginseng [53]. The cytotoxic effect of ginseng was reported to be via apoptosis [55] and autophagy induction [56]. Moreover, other properties such as the antiangiogenic and anti-inflammatory effects were reported [53] (Table 1, Table 2 and Table 3).

The steps from in vitro to in vivo testing and accessing clinical trials requires time and multiple resources. Based on technological advances, the time required for toxicological testing, a key step, has been reduced by methods developed by the Organization for Economic Co-operation and Development (OECD) and by the correlation of high-speed screening, biological phenotyping and integration with computer modeling in a new approach to the toxicological system [57]. In vitro studies require simpler procedures compared to in vivo and the achievement of the dose with a therapeutic effect of the dose is the basis for the implementation of clinical trials [58]. Unfortunately, the access of a molecule in the clinical trial phase is dependent at this stage mainly on the financial aspects, and most studies being stopped due to lack of funds. Additionally, favorable preclinical results usually do not translate to success in clinical trials [59] and relatively few of the actual isolated natural products are developed into clinically effective drugs [4].

## 3. Natural Compounds Effective as Chemopreventive Agents

Chemoprevention is defined as a pharmacological intervention [10] and several compounds encompassing those from natural sources, such as plants, fruits and vegetables, to synthetic molecules are able to inhibit, delay or reverse the process of tumorigenesis [4] and halt the risk of cancer recurrence [90] by blocking carcinogenic agents, increasing the capacity of the DNA repair system or acting directly against cells that carry DNA modifications by decreasing cell cycle speed or hindering events necessary for tumor spreading through metastasis [7]. It usually centers on the identification of agents that specifically impact early stages of cellular transformation [8] and addresses to people who have already developed cancer and also to those having a predisposition to any type of cancer [7]. Phytochemicals act in cancer prevention via direct and indirect strategies. The direct mechanisms include cell cycle arrest, autophagy and apoptosis, while reversing adverse epigenetic regulations, modulating miRNA expression, promoting the expression of phase II detoxification enzymes, balancing inflammation responses, aligning the circadian rhythm, and modifying gut microbiota all belong to the category of indirect chemopreventive strategies [91]. Taking into consideration the fact that the assault of free radicals causes DNA damage and alteration, phytochemicals exerting antioxidant activity could have a great contribution to cancer prevention [91,92].

In contradistinction to synthetic compounds, the naturally occurring agents have been presumed to be safer due to their presence in diet, wide availability and tolerability [10]. The identification of chemopreventive agents was possible from three main sources: (I) observations that populations presenting specific dietary habits have lower incidence of cancer development; (II) epidemiological studies or clinical tests of some drugs that show decreased number of cancers in some study populations as a secondary effect and (III) laboratory studies highlighting the inhibitory activities of some molecular entities on cancer cell cultures [7]. Phytochemicals have been found to have a wide range of cellular effects, and their chemopreventive activities can be attributed to their capacities to prevent carcinogens from reaching targeted sites, support detoxification of highly reactive molecules, enhance innate immune surveillance, improve the elimination of transformed cells or have several impacts on intrinsic DNA repair mechanisms by influencing tumor suppressors or inhibiting cellular proliferation pathways [8]. Other mechanisms involved in chemoprevention are direct inactivation of carcinogens either acting as free radical scavengers or inducing enzymes involved in scavenging, suppression of inflammatory events, induction of cell death through autophagy or apoptosis and inhibition of the epithelial-mesenchymal transition [7].

**Figure 8:**
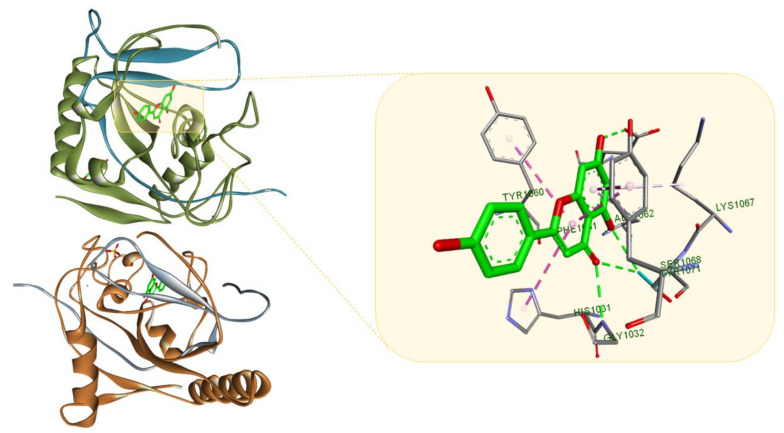
Apigenin (light green) in in complex with TNK2 (PDB ID: 4HKK); the flavone interacts with the surrounding amino acid residues through multiple HBs (green dotted lines) and several hydrophobic interactions (purple dotted lines). Protein-ligand representation was achieved using Biovia Discovery Studio 4.1 (Dassault Systems).

Plants are providers of endless supplies of secondary metabolites that are increasingly exploited against various cancers, among which the research on flavonoids has led to major developments in anticancer drug discoveries. The efficacy of flavonoids in chemoprevention highly depends on their pharmacokinetics and capacity of reaching to the biological site of action, as well as on their chemical structure. Moreover, flavonoids are characterized by a high availability, since they are abundantly present in our diet, such as fruits, vegetables, teas and wine, being gifted with a strong antioxidative potential, estrogenic regulatory effect, antimicrobial activity and ability to inhibit several points in cancer progression including invasion, metastasis and angiogenesis [9]. Some flavonoid compounds showing great potential in cancer prevention are apigenin, quercetin, luteolin and genistein. Apigenin is a natural phenolic compound [90], a flavone widely found in medicinal species such as *Lycopodium clavatum* L., as well as in some vegetables, including *Petroselinum crispum* L. and *Apium graveolens* L. Pharmacologically, it has been reported as a beneficial compound in DNA protection against UVB-induced damage via different mechanistic approaches: removal of the cyclobutane rings, inhibition of ROS generation and down-regulation of NF-κB. Moreover, apigenin possesses anti-inflammatory and antiproliferative properties [9]. By inhibiting the COX-2 expression, inducible nitric oxide synthase (iNOS) and nitric oxide (NO) generation, apigenin suppresses the inflammatory-triggered breast carcinogenesis and proliferation [90]. This phytocompound is also responsible for the inhibition of the Wnt/β-catenin signaling pathway, a key signaling cascade that participates in pivotal biological processes. Dysregulation of this pathway is frequently associated with numerous diseases including cancer [93]. The proposed mechanism for this particular activity suggests that apigenin inhibits tankyrase 2 (TNK2), an enzyme responsible for Wnt signaling pathway activation, telomere length and vesicle trafficking [93]. The study suggested that apigenin as well as other flavones target the nicotinamide binding site within TNK2 [94]. Figure 8 depicts the interactions formed by apigenin within the TNK2 binding domain. Apigenin manifests a potent activity on the endocrine function, acting as a progesterone agonist by binding to progesterone receptor and as an inhibitor of the autocrine-paracrine regulated metastatic processes, including angiogenesis, migration, invasion and adhesion through epithelial-mesenchymal transition reversal [90]. Regarding its activity on cancer cells, apigenin proved to be a pro-apoptotic compound [9].

Luteolin, a flavone found in *Salvia tomentosa* Mill., has been shown to delay or block the development of cancer cells, protect DNA from carcinogenic stimulus and induce apoptosis even in multidrug-resistant cancer cells by ROS generation, DNA damaging, activation of Ataxia Telangiectasia and Rad3-related/Checkpoint kinase 2/p53 (ATR/Chk2/p53) signaling, depletion of antiapoptotic proteins and inhibition of NF-κB signaling and of p38 [9]. Furthermore, luteolin possesses a progesterone antagonist activity, which can inhibit tumor growth through various targets in antioxidant, antitoxic, antiproliferative and pro-apoptosis pathways [90].

Genistein is an isoflavone and a natural dietary polyphenol commonly found in soybean (*Glycine max* L.) [9,90]. It has been reported to manifest a photoprotective effect against UVB irradiation, as well as an agonistic behavior on estrogen receptors. Thus, genistein can reactivate the estrogen receptor α (ERα) expression by remodeling chromatin structure in the ERα promoter and prevents tumorigenesis in ERα-negative breast cancer, which is clinically aggressive and does not respond to conventional estrogen target therapies [90]. By inducing alterations in the endocrine signaling, phenolic compounds such as apigenin, luteolin, quercetin and genistein may exert profound effects on chemoprevention and chemotherapy of hormone-dependent and independent breast cancers [90].

Curcumin might be a potential chemopreventive agent by modulating gene expression through epigenetic mechanisms [7] and some of the mechanisms involved in the effect on tumor cells have been highlighted in Table 1 and Table 2. Several studies identified that resveratrol exerts antitumor and cancer preventive effects through regulation of disease-specific molecular targets, affecting multiple stages of tumor initiation and proliferation [8]. This dietary polyphenol may also modulate the hormonal metabolism. A high intake of resveratrol is associated with lower risk of breast cancer, and lower levels of androgen precursors including androstenolone, could bring some benefits in the case of benign prostatic hyperplasia and cancer growth [8].

Betulin (lup-20(29)-ene-3β,28-diol) is a pentacyclic triterpenoid with a lupan-like structure [95]. Pentacyclic triterpenoids are active phytochemicals [96] exerting a wide spectrum of biological activities (e.g. antiviral, anti-inflammatory, anticancer) [97]. Betulin can act not only as a tumor cytotoxicity inducer [97], but also has a great potential to suppress tumor formation [96]. The role of betulin in cancer prevention and treatment results from its pro-apoptotic, antiangiogenic, and antioxidant properties [96,98], as well as its ability to alter cytomorphological features in cancer cells and to induce necrosis [95]. Betulin manifests an important scavenging effect against several radicals such as 2,2’-azinobis-(3-ethylbenzothiazoline-6-sulfonic acid) (ABTS), 1-diphenyl-2-picryl hydrazyl (DPPH), NO, and superoxide anion [99] and shows immunostimulatory activity [100].

Phytochemicals are essential in the prevention of cancer occurrence and development, bringing a myriad of benefits for human health. The optimum intake of natural chemopreventive agents might be reached through diets that are rich in fruits and vegetables, as well as through the help of highly-standardized supplements containing the active compounds.

## 4. Natural Compounds as Sensitizers in Drug Resistance

Cancer is one of the most impactful diseases of the 21st century [101] and a major cause of death globally [14], each type having its own molecular fingerprint [102]. Chemotherapy is one of the main options for cancer treatment, using molecules capable of inhibiting proliferative signaling pathways, replicative immortality mechanisms and angiogenesis or inducing apoptosis of tumor cells [103,104]. It usually consists of either single therapy, or a combination of classical treatments, depending on several factors such as cancer type and underlying biological conditions in patients [14]. Monotherapy has been a traditional approach in treating diseases that is widely based on the pharmacological dogma of “one drug-one target”. However, combined therapies proved to be much more efficient than single-drug-based treatments. The most relevant characteristic in combined therapies is the synergistic effect [14], which can be described as an increase in efficacy for a combination of components when compared with a single one [15]. Even though anticancer research and drug discovery are continuously increasing the therapeutic arsenal [102], chemotherapy fails due to adverse reactions, drug resistance and target specificity [14].

Drug resistance remains one of the most challenging aspects of chemotherapy, being a consequence of several factors depending on therapy, population of cancer cells and host environment. Vasan el al. framed the main determinants of drug resistance as follows: therapeutic pressure, tumor burden, growth and heterogeneity, physical barriers, immune system, and undruggable genome [105]. When cancer drug resistance occurs, higher doses need to be applied in order to achieve a similar tumoricidal effect as the initial dosage, which enhances the risk of severe side effects. The molecular mechanisms of chemoresistance are drug influx or efflux, DNA damage repair, cell death inhibition, drug inactivation, epithelial-to-mesenchymal transition, drug target alteration, NF-κB and STAT-3 activation [15,103]. Drug efflux, the process through which cancer cells pump chemotherapeutics out of the cells using multidrug resistance proteins, is the most important mechanism [15,106]. Chemosensitization by definition refers to the potentiation of the tumoricidal effect of chemotherapeutic drugs [15]. Therefore, chemosensitizers that are able to accumulate preferably within tumor site and potentialize systemic conventional therapeutic effects could represent potential candidates for fighting drug resistance [106]. According to previous studies, plant constituents can improve the effectiveness of conventional chemotherapy by increasing the residence time of chemotherapeutics in tumor cells, inducing cell death by up-regulation of pro-apoptotic targets, promoting DNA damage or regulating the expression of altered and unaltered drug targets. These mechanisms enhance the cytotoxic effect of anticancer drugs, promoting a synergistic effect even in the cells with acquired resistance [101,104]. Therefore, they can increase drug efficacy at lower dose levels and thus, reducing its toxicity [106]. Natural agents such as phenolic derivatives, flavonoids, alkaloids, carotenoids, terpenoids, quinones, saponins and steroids are among the potential chemosensitizers [101,103].

For instance, it has been noticed that **resveratrol** can sensitize resistant cells to chemotherapeutic agents via multiple mechanisms of action. To mention but a few, resveratrol was able to sensitize human cancer cell lines such as leukemia, neuroblastoma, glioblastoma and breast, prostate and pancreatic carcinomas to doxorubicin, cytarabine, actinomycin D, taxol and methotrexate by down regulating surviving expression and increasing apoptosis. It was shown as well that resveratrol enhances the chemotherapeutic potential of doxorubicin in chemoresistant B16 melanoma cells through up-regulation of the p53 tumor suppressor gene and synergistically promotes the 5-fluorouracil-mediated apoptosis [103]. Another study referring to resveratrol, highlights its potential in enhancing the effects of cisplatin on the inhibition of human non-small cell lung cancer cell proliferation via cell apoptosis induction, depolarization of mitochondrial membrane potential, release of cytochrome c and regulation of Bcl-2 and Bax expression [107]. Two in vitro studies revealed that the micellar co-delivery of resveratrol and quercetin or curcumin influences the doxorubicin treatment of ovarian cancer. The results, in both cases, suggest that the combination of natural compounds mitigates doxorubicin-induced cardiotoxicity through reduction in apoptosis and ROS, while providing a secondary benefit of acting as a chemosensitizer [108,109].

Other studies reveal that the association between **curcumin** and 5-fluorouracil causes synergistic inhibition of growth and induction of potent apoptosis in resistant breast and gastric cancer cell lines in vitro, through NF-κB downregulation, reducing the toxicity of the drug as well [106,110]. Sreekanth et al. observed that the combined treatment of curcumin and paclitaxel induced a synergistic reduction in the cervical tumor incidence and volume in animals, compared with the individual treatments of paclitaxel or curcumin [111]. Suzuki and colleagues showed, both in vitro and in vivo, the ability of the soy-derived isoflavone, genistein, to potentiate the antitumor effect of 5-fluorouracil in pancreatic cancer cells. The mechanisms of action, according to authors, are induced apoptotic, as well as autophagic cell death in cancer cells [112].

## 5. Efficient Platforms for the Incorporation and Delivery of Natural Compounds

Phytochemicals have demonstrated the same validity as synthetic or semisynthetic compounds in drug discovery [102]. Regarding cancer management, they can be used in a versatile manner as chemotherapeutic, chemopreventive or chemosensitizer agents [101].

Although some natural compounds have unique antiproliferative effects, their use in clinical practice is not possible due to their toxicity or physicochemical properties, which limit their bioavailability. This is the reason why there are many of their derivatives in current clinical studies, as the modification of chemical structures increases the anticancer action and selectivity, improves the pharmacokinetic properties and decreases the side effects of natural compounds [6].

However, drawbacks such as poor solubility, limited dissolution rate, instability in extreme pH values, scarce absorption, high metabolization rate and fast excretion process, resulting in low bioavailability and less or no therapeutic effect, limit their use in clinical practice [101,113]. According to scientific literature reports, one approach to overcome these limitations is loading the natural compounds into different types of delivery systems such as liposomes, micelles, polymeric nanoparticles, dendrimers or inorganic nanoparticles [114]. Drug delivery refers to the use of a tool or vehicle able to carry a therapeutic agent and release it at a specific location [114]—for instance, the tumor site. These innovative carriers are able to optimize some unfavorable physicochemical features of the natural active compounds, while enhancing their pharmacological activity and bioavailability by enabling them to cross the cellular membranes of target cells [113,114]. Different forms of encapsulation have been developed throughout the last decades in order to deliver natural anticancer agents.

To name but a few examples, Li and collaborators [115] evaluated the preclinical antitumor effect of curcumin-loaded liposomes in colorectal cancer, compared to a standard chemotherapeutic, oxaliplatin. The curcumin-loaded liposomes were obtained using as lipids: 1,2-dimyristoyl-sn-glycero-3-phosphocholine, 1,2-dimyristoyl-sn-glycero-3-phosphoethanolamine-N-[methoxy-(polyethylene gly-col)-2000], and 1,2-dimyristoyl-sn-glycero-3-[phospho-rac-(1-glycerol)] (sodium salt) in a 10:1 ratio (lipids : curcumin). Their study establishes that liposomal curcumin induced a dose-dependent growth inhibition and apoptosis in vitro, as well as a significant tumor growth inhibition and antiangiogenic effect in vivo. Liposomal curcumin demonstrated a greater antiproliferative effect than oxaliplatin in LoVo cells and an equivalent effect in Colo25 cells [115,116]. Zhao et al. investigated the ability of peptide and sucrose liposomes to enhance the physicochemical properties and the effect of resveratrol against breast cancer, both in vitro and in vivo. According to their results, this formulation was exceptionally efficient in inhibiting the proliferation of breast cancer cells MCF-7 (IC_50_ = 20.89 µmol/L) and blocking tumor growth completely at a dosage of 10 mg/kg in vivo. Liposomal resveratrol was capable of inducing a greater cell apoptosis and less toxicity in mice than the compound alone and manifested a selective cytotoxic activity on cancer cells [117]. In another study, Sanna et al. developed polymeric (−)-epigallocatechin-3-gallate—encapsulated nanoparticles targeted with small molecular entities and evaluated their efficacy against prostate cancer in preclinical studies. The encapsulation led to an enhanced bioavailability and antiproliferative activity compared to the free EGCG, limiting its unwanted toxicity [118]. Lazzeroni and collaborators conducted a pilot "window-of-opportunity" presurgical trial in order to test the effect of a lecithin formulation of green tea extract in twelve women diagnosed with breast cancer. Patients received 300 mg of extract daily for 4 weeks prior to lumpectomy or mastectomy. Firstly, their results indicated a suitable bioavailability of green tea polyphenols and EGCG in breast tumor tissue, suggesting that the formulation with lecithin is able to improve the absorption of EGCG without compromising its safety. Secondly, they noticed a positive correlation between free EGCG plasma levels and the decrease in the tumors, indicating its influence on cell proliferation in breast cancer tissue [33]. Rastegar and colleagues synthesized a novel magnetic platform based on β-cyclodextrin for the co-delivery of doxorubicin and curcumin, able to release the compounds in a pH-dependent manner. Their drug delivery system was efficiently internalized by human breast carcinoma cells and accumulated into the tumor via external magnet guidance. Regarding the anticancer activity, a superior cytotoxicity and reduction in relative tumor volume size compared to the control group were obtained [119]. In cancer therapy, paclitaxel-based liposomes have been approved since the early 2000s, followed by the introduction of paclitaxel nanoparticles into the market [5], effectively paving the way for clinical trials of these efficient delivery platforms.

Recently, melanin—the ubiquitous biopolymer and pigment that is widely distributed in various parts of living organisms, including skin, hair and eyes [120]—came out as a promising tool for biomedical applications [121]. The exploration of melanin nanoparticles has substantially broadened new horizons in the development of novel drug delivery systems, especially due to their unique properties, which are missing in the case of conventional platforms such as liposomes, micelles, polymeric nanoparticles, and so forth [121]. The advantages include superior biocompatibility and biodegradability, intrinsic photoacoustic and photoprotective properties, radical scavenging capacity and ability to form chelates with drugs or metal ions [121,122]. Bioactive molecules can either bind to melanin via π–π stacking, hydrogen bonding and van der Waals interaction, or simply be conjugated onto the surface or encapsulated within the polymer matrix of the nanoparticles [120].

For instance, Wang et al. developed doxorubicin-loaded melanin nanoparticles to enhance chemotherapy against drug-resistant thyroid cancer. The cellular uptake and therapeutic efficacy were significantly higher than those induced by the same amount of free doxorubicin, reaching a cell viability of 34.6 ± 5.4%, at the highest concentration of 160 mg/L. Moreover, no significant cytotoxicity to normal cells was noticed, proving the biocompatibility of the nanoparticles [123]. Another research team investigated the properties and activity of polyethylene glycol (PEG)-conjugated melanin nanoparticles loaded with varied concentrations of doxorubicin. Melanin nanoparticles showed a high drug loading capacity, and the release of doxorubicin was primarily diffusion controlled. Regarding their in vitro activity, the loaded nanoparticles manifested no toxic effect on mouse fibroblasts, which indicate a good cytocompatibility, but selectively inhibited the proliferation of human breast cancer cells. Moreover, the PEGylated melanin nanoparticles, according to authors, might serve as carriers for the prolonged release of anticancer drugs [122]. Song and colleagues developed complex silver-decorated melanin-like polydopamine/mesoporous silica composites loaded with curcumin and evaluated their antibacterial and antitumor properties. Their delivery platform proved to be highly biocompatible and permitted a ROS or pH-dependent release due to the blockage of π−π and/or hydrogen bonding between the polydopamine layer and curcumin/silver. Notably, a pronounced chemotherapeutic effect on human cervical cancer cells and taxol-resistant non-small cell lung cells was observed [124]. The studies performed for the development and characterization of silver nanoparticles functionalized with betulin have highlighted cytotoxic and antiproliferative properties on murine melanoma cells in vitro (high selective toxicity and a dose-dependent pro-apoptotic effect) and an antimetastatic potential (changes of tumor cells morphology, from fusiform to a less aggressive epithelial shape) in an animal model in vivo [98]. Additionally, the conjugation of gold nanoparticles with betulin showed promising results in terms of cytotoxic and apoptotic activity on murine and human melanoma cells [125]. Betulin-based nanoemulsion proved to be a true candidate with antitumor effect in studies involving surface enhanced Raman spectroscopy (SERS) for the analysis of an animal model of induced skin cancer, validating by histological examination and chemometric methods, its beneficial effect [126].

In recent years, heat-based therapeutic strategies have gained considerable attention. Studies aimed at improving the current therapy of breast cancer with the help of liposomal nano-platforms with incorporated bioactive agent, sensitive to heat, are promising. Betulinic acid-loaded magnetoliposomes have been shown to have an enhanced antitumor activity based on impairment of mammary adenocarcinoma cells, correlated to the enucleation process and DNA fragmentation [127]. Several examples of the delivery systems developed for the discussed bioactive molecules are presented in Table 4.

Even though natural compounds present a high potential in treating several types of cancer, their pharmacokinetic characteristics hinder the clinical use. The most promising solution, in this case, seems to be the use of drug-delivery platforms which are able to improve the bioavailability and the antitumor activity of phytochemicals, while reducing their side effects.

## 6. Conclusions

Natural compounds are still considered an inexhaustible source of models in finding new active chemotherapeutic agents, while providing the basis for the discovery of new structures that can be approved as therapeutical agents for a variety of human diseases. Even though a considerable number of natural compounds prove therapeutical efficacy in preclinical studies their number decreases dramatically until they reach the clinical trial phase. The selection of the most appropriate in vitro and in vivo models that attest the effectiveness of the natural compounds and ensure their inclusion in clinical trials, remains a challenge for researchers. To overcome these liabilities, there should be proposed alternative in vitro and in silico methods that can significantly reduce the time and costs required for the in vivo studies and to concentrate the resources, especially the financial ones, for the investigation of biocompounds in clinical trials. In most cases, the effectiveness of natural compounds is limited by their low bioavailability. Thus, the researchers must focus their investigation not only on the efficacy of the compound, which is of great interest, but also on the drug delivery systems able to overcome its pharmacokinetic issues, as well as on the study of the suitable derivatives which bring several benefits regarding biological availability and efficacy.

## Figures and Tables

**Table 1:** Relevant in vitro studies, based on natural compounds with antitumor properties, subsequently correlated with in vivo studies and clinical trials.

Natural Compound	Cancer Type	Cell Line (s)	Active Concentrations	Findings	Cell Death Type	Ref.
**Resveratrol**	Breast cancer	4T1	IC_50_ = 93 µM (72 h)	Cell cycle inhibition; S-phase arrest; ↑ cell apoptosis rate	Apoptosis	[60]
	Osteosarcoma	MNNG/HOS	IC_50_ = 20.57 μM (48 h)	Cleavage of PARP, and caspase-3; ↑ Bax; ↓ Bcl-2 and Bcl-xL; JAK2/STAT3 pathway inhibition	Apoptosis	[61]
		MG-63	IC_50_ = 28.56 μM (48 h)			
	Cervical cancer	HeLa	IC_50_ = 188 ± 19 µM (48 h)	↑ content of LAMP1, Atg7, LC3B, PINK1 and PARK2 proteins; ROS overload; ↓ glycolysis; ↓ oxidative phosphorylation protein contents and fluxes	Autophagy	[62]
	Colorectal cancer	SW480	5 µM	inducing cell cluster formation; ↓ cell viability	Apoptosis	[63]
		HCT116				
**Curcumin**	Melanoma	A375	IC_50_ = 25 µM (24 h)	inhibition of cell invasion; G2/M phase cell-cycle arrest; suppression of the AKT, mTOR and P70S6K activation	Autophagy	[64]
		C8161	IC_50_ = 15 µM (24 h)			
	Breast Cancer	MCF-7	1–25 μg/mL (72 h)	↓ phosphorylation of Akt and MAPK; ↓ HER-2 oncoprotein; ↓ NF-κB	-	[65]
		MDA-MB-231				
		BT-474				
		SK-BR-3-hr				
	Cervical Cancer	HeLa	12–14 µM (48 h)	DNA damage and fragmentation; chromatin condensation; ↑ amounts of p-ATM, p-ATR, p53, MDM2, BRCA1, DNA-PK, MDC1 and p-H2A.X, PARP and MGMT proteins	Apoptosis	[66]
	Colon Cancer	SW480	1–50 µM (24 h)	inhibition of the cellular invasive activity; ↓ uPA and MMP9 expression; ↓ NF-κB activation	-	[67]
		LoVo				
**EGCG**	Colorectal Cancer	DLD-1	20–60 µM	↓ number and size of cell sphe-roids;inhibition of the Wnt/β-catenin pathway; ↓ pro-tein levels of Cyclin D1 and PCNA; ↓ Bcl-2; ↑ Bax, Caspase-8, Caspase-9, and Caspase-3	Apoptosis	[68]
		SW480				
	Lung Cancer	A549	IC_50_ = 36.0 μM (72 h)	↓ survival rate; loss of the adhe-sion ability; ↓ Bcl-xL	Apoptosis	[69]
	Breast Cancer	MDA-MB-231	≥ 75 μM (24 h)	↓ expression of β-catenin, p-Akt, and cyclin D1; inactivation of the β catenin signaling pathway; ↓ cell proliferation; disrupted adherence junction formation	-	[70]
**Quercetin**	Pancreatic Cancer	LNCaP DU-145 PC-3	40 μM (24, 48, and 72 h)	modulation of ROS production and mitochondrial membrane potential; interference with MAPK, Akt, and NF-κB signaling pathways	Apoptosis Necrosis	[38]
	Cervical Cancer	HeLa	EC50 = 100 μM (24 h)	↑ expression of caspases and pro-apoptotic genes; DNA fragmentation; ↓ cell migration; G2-M cell cycle arrest; blockage of the PI3K, MAPK and WNT pathways	Apoptosis	[37]
	Colorectal Adenocarcinoma	Caco-2	IC50 = 35 μM (24 h)	NF-κB pathway inhibition; ↑Bax; ↓Bcl-2 increased cell membrane permeability; nuclear condensation	Apoptosis	[71]
**Rutin**	Colon Cancer	HT-29	100 and 200 μM (24 h)	↑ cleaved caspases-3, -8 and -9; ↓ Bcl 2; ↑ Bax; cell shrinkage; chromatin condensation; rounding, blebbing and an increased density of apoptotic bodies	Apoptosis	[72]
**Betulinic acid**	Melanoma	B164A5	10 mM	G0/G1 cell cycle arrest; ↓ cell proliferation	Apoptosis	[46]
	Ovarian Cancer	A2780	IC50 = 44.47 μM (24 h)	↑ levels of cleaved caspase-8, -3, -9; ↑ Bax; ↓ Bcl-2; nuclear Condensation	Apoptosis	[73]
	Colon Cancer	HCT116 SW480 DLD-1	≥ 5 µg/mL (48 and 72 h)	↑ Bax; ↑ cleaved caspase-3; ↑ ROS production; ↓ Bcl-2; ↓ mitochondrial membrane potential	Apoptosis	[74]
**Artemisinin** **(Artesunate)**	Breast Cancer	MDA-MB-231	25, 50 and 100 μM (48 h)	↓ Bcl-2; ↑ Bax; G2/M-phase arrest; ↓ Cyclin-B1 and Cyclin-D1; agglutinated heterochromatin; degenerated mitochondrial vacuoles; nuclear swelling; ↓ number of intracellular organelles	Apoptosis Autophagy	[50]
	Colon Cancer	HCT116	40 and 80 μM (24, 48, and 72 h)	Cell elongation; membrane foaming; nuclear condensation and fragmentation; chromatin shrinkage; ↓ Bcl 2 and Bcl xL; ↑ Bax; ↑ cleaved procaspase-3 to active caspase-3; ↑ levels of beclin 1, LC3 I/II, and Atg5; ↑ complexation of Atg12-Atg5	Apoptosis Autophagy	[51]
	Cervical Cancer	ME-180	300 and 500 μM (48 and 72 h)	↓ Telomerase activity; ↓ hTERT and hTR expression; ↓ E6 and E7 oncogenes; chromatin condensation; ↑ p53 expression	Apoptosis	[75]
**Ginseng extract**	Breast Cancer	MCF-7	100–400 μM (24 h)	↓ Bcl-2; ↑ Bax, cytochrome c, and cleaved caspase-3; ↑ ROS production	Apoptosis	[55]
	Lung Cancer	A549	50–100 μM (48 and 72 h)	Punctate cytoplasmic expression of LC3, Beclin-1 and ATG5; G2/M phase arrest; ↑ expression of LC3-II; ↑ p-Akt; ↓ mTOR-4EBP1	Autophagy	[56]

**Table 4:** Drug delivery systems for the efficient encapsulation and enhanced anticancer effect of natural compounds.

Natural Compound	Drug Delivery System	Findings	Ref.
**Resveratrol**	Peptide and Sucrose Liposomes	Prolonged drug-release in vitro; Breast cancer (MCF-7) cells growth inhibition (IC50 = 20.89 μmol/L); Apoptosis; Tumor growth inhibition in mice bearing breast cancer (Dose = 10 mg/kg)	[128]
	Sulfobutylether-β-cyclodextrin	Improved stability; Longer half-life; Significant cytotoxic potential against non-small cell lung cancer; Suitable for pulmonary delivery	[117]
	Gold Nanoparticles	Optimal cellular uptake; Superior cytotoxic effects on breast, pancreatic, and prostate cancers	[129]
**Curcumin**	Silver-Decorated Melanin-like Polydopamine/Mesoporous Silica Composites	Improved chemotherapeutic efficiency against human cervical cancer cells (HeLa) and Taxol-resistant non-small cell lung cells (A549/TAX); Desirable biocompatibility; Low hemolytic activity	[124]
	Liposomes	Potent cytotoxic effect against human MiaPaCa pancreatic cancer cells (IC50 = 17.5 µM); suppression of tumor growth in tumor-bearing nude mice (Dose = 20 mg/kg); Potent antiangiogenic effect	[130]
	Cyclodextrins	Enhanced delivery; Improved therapeutic efficacy against lung cancer in vitro and in vivo	[131]
**EGCG**	Deformable Liposomal Formulation	Increased drug-release; Optimal formulation for topical delivery in skin cancer prevention	[132]
	Folic acid and polyethylene glycol (PEG)-modified Nanoparticles	Inhibition of MCF-7 cells proliferation in a dose-dependent manner; Enhances targeting ability and efficacy of the drug	[133]
**Quercetin**	Lipid Nanoparticles	Sustained release; Inhibition of the MCF-7 breast cancer cells growth; Increased ROS production; Increased apoptotic and necrotic indexes in MCF-7 cells	[134]
	Polymeric Nanoparticles	Enhanced efficacy in cancer therapy; Reduced tumor volume in breast and lung-bearing mice;	[135]
**Rutin**	Proniosomal Gel	High biocompatibility of the gel on the 3D reconstructed human epidermis; Lack of irritant and phototoxic potential; Preferential cytotoxic effect of the drug on melanoma cells (IC_50_ = 8.601 µM)	[136]
	β-cyclodextrins and hydroxypropyl-β-cyclodextrins	Increased antioxidant activity; Antiproliferative and pro-apoptotic effect against B164A5 murine melanoma cells	[137]
**Betulinic acid**	Gamma-cyclodextrins	Improved antiproliferative activity in vitro on metastatic and non-metastatic B164A5 melanoma cells; G0/G1 cell-cycle arrest; Reduced in vivo tumor development	[46]
	Magnetoliposomes	Enhanced antitumor activity when breast adenocarcinoma MDA-MB-231 cells and a microtubule assembly modulatory activity under hypertermic conditions;	[127]
	Silver Nanocolloids	Augmented anticancer effect against lung A549 and liver HepG2 cancer cell lines; Cell type- and time-dependent cytotoxic effect	[138]
**Artemisinin** **(Artesunate)**	Chitosan Magnetic Nanoparticles	Enhanced accumulation of nanoparticles in the 4T1 breast tumor tissues of BALB/c mice model	[139]
	pH-Responsive Lipid Nanoparticles	Inhibition of the breast cancer cells growth; down-regulation of the anti-apoptotic protein survivin, and cyclin D1; down-regulation of the oncogenic proteins HER2 and HER3; Reduced expression of the epidermal growth factor receptor (EGFR or HER1)	[140]
**Ginseng**	Ginsenoside Rb1/Protopanaxadiol Nanoparticles	High encapsulation efficiency, drug loading capacity, and slow release kinetics; Lack of hemolytic effect; Superior in vitro anticancer activity on murine Lewis lung carcinoma	[141]
	Ginsenoside-based multifunctional liposomal delivery system	Successful delivery of the bioactive combination drugs and internalization into gastric cancer cells; Suppressed gastric cancer tumor growth	[142]

**Table 2:** In vivo studies achieved after the successes recorded in vitro and promulgated in clinical trials.

Natural Compound	Cancer Type	Animal Model	Dose/Administration	Findings	Ref.
**Resveratrol**	Breast cancer	MDA-MB-231 cells xenograft model in female athymic mice	intraperitoneal administration of 25 mg/kg/day of resveratrol (ethanolic solution) for 3 weeks	↓ tumor size; ↑ apoptotic index; ↓ angiogenesis	[76]
	Lung cancer	A/J mice with 4-[methyl(nitroso)amino]-1-(3-pyridinyl)-1-butanone-induced lung carcinogenesis	intranasal administration of ~60 mg/kg three times a week for 25 weeks	↓ tumor size (27%); ↓ tumor volume (45%) chemopreventive properties in the chemically-induced lung cancer	[77]
	Melanoma	B16F10 cells xenograft model in female C57BL/6 mice	intravenous administration of 0.5 mg/kg of resveratrol (PBS solution) 6 times on days −2, 0, 2, 4, 6, and 8	inhibition of tumor growth and metastasis; ↑ NK cell activity	[78]
**Curcumin**	Breast Cancer	HER-2-overexpressed BT-474 xenograft mod-el in female athymic nude mice	intraperitoneal administration of curcumin dissolved in 0.1% DMSO at a dose of 45 mg/kg twice/week for 4 consecutive weeks	↓ tumor volume (by 76.7%)	[65]
	Cervical Cancer	Human cervical cancer HeLa cells xenograft model in nude mice	intraperitoneal administration of curcumin at a dosage of 50, 100 and 200 mg/kg, once/two days for 20 days	↓ tumor volume and mass; tumor inhibition rate percentages were 1.7%, 31.1% and 39.6% for curcumin 50, 100 and 200, respectively	[79]
	Colon Cancer	HCT-116 Cells Xeno-graft model in female homozygous ICR SCID mice	500 mg/kg body by gavage every day for 3 weeks	↓ growth of p53-positive (wt) and p53-negative colon cancer HCT-116 cells; ↓ proliferation and ↑ apoptosis accompanied by the attenuation of NF-κB activity; synergistic effect with resveratrol	[80]
**EGCG**	Colorectal Cancer	Orthotopic colorectal cancer xenograft model in BALB/c nude mice	intragastrical administration of 5, 10 and 20 mg/kg of EGCG once daily for 14 days	↓ tumor volume; ↑ apoptotic rates for EGCG 5, 10, and 20 mg/kg (38.04%, 51.87%, 52.27%, and 54.33%)	[81]
	Lung Cancer	A549 cells xenograft model in BALB/c nude male mice	0.05% EGCG solutions in DMSO administered in drinking water daily for 13–21 days	↓ tumor growth; ↓ angiogenesis and CD34 positive vessels	[82]
	Breast Cancer	MCF-7 cells xenograft model in female CB-17 severe combined im-munodeficient mice	100 mg/kg of EGCG dissolved in 100 μL water every 2 days by oral gavage	↓ tumor growth; ↓ expression of miR-25; ↓ Ki-67 and ↑ pro-apoptotic PARP expression	[83]
**Quercetin**	Pancreatic Cancer	MIA PaCa-2 cells Orthotopic xenograft model in nude mice	1% quercetin -supplemented diet for 42 days (Oral administration)	↓ tumor volume and weight; ↓ tumor cell proliferation; ↑ apoptotic cell death	[84]
**Rutin**	Colon Cancer	SW480 cells xenograft in nu/nu mice	daily intraperitoneal administration of rutin at different doses (≤ 20 mg/kg) for 32 days	↓ tumor volume and weight; ↑ mean survival time by 50 days; ↓ VEGF serum levels (by 55%)	[85]
**Betulinic acid**	Colorectal cancer	HCT116 cells xenograft model in mice	daily intraperitoneal administration of 10 and 20mg/kg/day of betulinic acid for 21 days	↓ tumor growth; ↓ number of Ki67-positive and MMP-2-positive cells; ↑ cleaved caspase-3-positive cells	[74]
**Artemisinin** **(Artesunate)**	Breast Cancer	4T1 cells xenograft model in female BALB/c mice	intraperitoneal injection with 100 mg/kg artemisinin dissolved in a 0.2% DMSO solution) daily, for 20 days	↓ Treg and MDSC expansion in the spleen and tumor; ↑ percentages of CD4 + IFN-γ + T cells; ↑ FN-γ and TNF-α	[86]
	Colorectal Cancer	CLY cells xenograft model in female athymic nude mice (Balb/c nu/nu)	intravenous administration of artesunate as follows: (1) intermittent large dose treatment (300 mg/kg; every 3 days, for 7 days) and (2)persistent small dose treatment (100 mg/kg; every day, for 20 days)	slow growth of tumor xenografts; ↓ physiological activity of tumor xenografts; delayed spontaneous liver metastasis.	[87]
	Cervical Cancer	HeLa cells xenografts in male BALB/c mice	intraperitoneal administration of 100 mg/kg/day artesunate for 7 days	↓ microvessel density; ↑ apoptosis; ↑ Cyclin B1 expression G2-M phase arrest; ↑ radio-sensitivity	[88]
**Ginseng**	Breast Cancer	MCF-7 cells xenograft model in female BALB/*c* athymic nude mice	intravenous administration of Ginseng extract (50 or 100 mg/kg), once a day for 4 weeks	↓ tumor weight; ↑ Bax, cleaved caspase-3, and cleaved PARP; ↓ Bcl-2	[55]
	Lung Cancer	LLC-1 cells xenograft model in male C57BL/6J mice	Asian Ginseng extract (0.25, 0.5, and 1 g/kg/day) daily administration as pretreatment (for 10 days) and treatment (for 20 days)	↓ tumor volume and mass; ↓ cell proliferative index; ↓ P-Stat3 and PCNA	[89]

**Table 3:** Relevant examples of natural compounds found in clinical trials (according to data from US National Library of Medicine – ClinicalTrials.gov).

Natural Compound	Identifier/Status	Cancer Type/Conditions	Title	Observations
**Resveratrol**	NCT00256334/Completed	Colon cancer	Resveratrol for Patients With Colon Cancer	Patients were randomly assigned to one of four dose cohorts: plant-derived resveratrol tablets (80 mg/day and 20 mg/day), Grape Powder dissolved in water and taken orally (120 g/day and 80 g/day).
	NCT02261844/Withdrawn (No funding)	Liver cancer	Resveratrol and Human Hepatocyte Function in Cancer	Resveratrol 1 g daily for 10 days Dietary Supplement: Resveratrol Resveratrol 1 g po x 10 days prior to liver resection
	NCT01476592/Completed	Neuroendocrine tumor	A Biological Study of Resveratrol’s Effects on Notch-1 Signaling in Subjects With Low Grade Gastrointestinal Tumors	5 g/day of resveratrol orally, in two divided doses of 2.5 g each without a break in therapy for a total of three cycles
	NCT00433576/Completed	Colorectal cancer	Resveratrol in Treating Patients With Colorectal Cancer That Can Be Removed By Surgery	STAGE II: Patients receive oral resveratrol on days 1–8 and undergo colorectomy on day 9.
	NCT00098969/Completed	Unspecified Adult Solid Tumor, Protocol Specific	UMCC 2003-064 Resveratrol in Preventing Cancer in Healthy Participants	This phase I trial is studying the side effects and best dose of resveratrol in preventing cancer in healthy participants.
	NCT03253913/Unknown	Lymphangioleiomyomatosis	Resveratrol and Sirolimus in Lymphangioleiomyomatosis Trial	Resveratrol 250 mg daily for the first 8 weeks, followed by 250 mg twice daily for the next 8 weeks, and then 500mg twice daily for the last 8 weeks.
	NCT04266353/Suspended (Due to COVID-19)	Breast cancer	Effect of Resveratrol on Serum IGF2 Among African American Women	Participants with receive resveratrol at 150 mg daily for 6 weeks
	NCT00578396/Unknown	Colon cancer	Phase I Biomarker Study of Dietary Grape-Derived Low Dose Resveratrol for Colon Cancer Prevention	-
**Curcumin**	NCT03980509/Recruiting	Breast Cancer	A "Window Trial" on Curcumin, the Active Compound in Turmeric, for Invasive Breast Cancer Primary Tumors	Curcumin (500 mg) will be orally administered twice a day, after each meal from the time surgical resection is scheduled until the night before surgical resection.
	NCT04294836/Not yet Recruiting	Cervical Cancer	Randomized Phase II Clinical Trial of Oral Turmeric Supplementation in Patients With Advanced Cervical Cancer	Curcumin administered in a dosage of 2000 mg daily, in association with cisplatin and radiotherapy for 16 weeks
	NCT02724202/Active, not recruiting	Colon Cancer	A Pilot, Feasibility Study of Curcumin in Combination With 5FU for Patients With 5FU-Resistant Metastatic Colon Cancer	Curcumin at a dosage of 500 mg twice/day will be orally administered for 2 weeks. Patients will continue on curcumin at the same dose for an additional 6 weeks while being treated with 3 cycles of 5-fluorouracil
**EGCG**	NCT02891538/Recruiting	Colorectal Cancer	A Pilot Study to Evaluate the Chemopreventive Effects of Epigallocatechin Gallate (EGCG) in Colorectal Cancer (CRC) Patients With Curative Resections	EGCG (highly purified and refined green tea extract-Teavigo™) administered at a dosage of 450 mg twice a day
	NCT01317953/Available	Lung Cancer	Phase I Study of Oral Green Tea Extract as Maintenance Therapy for Extensive-stage Small Cell Lung Cancer	Increasing doses of EGCG (400, 800, 1200, 1600 and 2000 mg) administered daily
	NCT00917735/Completed	Breast Cancer	Phase II, Randomized, Double-blind, Placebo-controlled, Study of the Efficacy of Green Tea Extract on Biomarkers of Breast Cancer Risk in High Risk Women With Differing Catechol-O-methyl Transferase (COMT) Genotypes	Oral administration of two Green tea extract capsules containing 51.7% EGCG, twice daily after breakfast and dinner for one year.
**Quercetin**	NCT01538316/ Unknown	Prostate Cancer	Clinical Trial on the Effectiveness of the Flavonoids Genistein and Quercetin in Men With Rising Prostate-specific Antigen	500 mg of quercetin supplement (+ vitamin C + folic acid + vitamin B3) administered daily over a period of six months, followed by genistein and placebo administration.
	NCT03476330/Recruiting	Squamous Cell Carcinoma	Quercetin Chemoprevention for Squamous Cell Carcinoma in Patients With Fanconi Anemia	Quercetin administered orally twice daily at an wheight-based adjusted dosage (maximum 4000 mg/day).
**Betulinic acid**	NCT00346502/Suspended	Dysplastic Nevus Syndrome	Phase I/II Evaluation of Topical Application of 20% Betulinic Acid Ointment in the Treatment of Dysplastic Nevi With Moderate to Severe Dysplasia	Daily application of the 20% betulinic acid ointment to the dysplastic nevi site for a period of four weeks.
**Artemisimin** **(Artesunate)**	NCT00764036/Completed	Breast Cancer	Prospective Open Uncontrolled Phase I Study of Compatibility, Safety&Pharmacokinetics of Artesunate, a Semisynthetic Derivative of Artemisinin From the Chinese Herb Artemisia Annua in Patients With Metastatic/Locally Advanced Breast Cancer	The administration of the drug was as follows: daily single oral doses of 100, 150 or 200 mg of artesunate, for 4 weeks.
	NCT03093129/Recruiting	Colorectal Cancer	Phase II Randomised, Double Blind, Placebo Controlled Trial of Neoadjuvant Artesunate in Stage II/III Colorectal Cancer in Vietnamese Patients	Daily administration of artesunate (200 mg) for 14 days.
	NCT04098744/Recruiting	Cervical Neoplasia	A Phase II Double Blind, Placebo-controlled, Randomized Trial of Artesunate Vaginal Inserts for the Treatment of Patients With Cervical Intraepithelial Neoplasia (CIN2/3)	Participants will receive three 5-day cycles of artesunate inserts, 200 mg per day, at weeks 0, 2, and 4.
**Rutin**	NCT00003365/Terminated	Colon Cancer	The Effect of Plant Phenolic Compounds on Human Colon Epithelial Cells	The administration of rutin was twice a day, for 6–10 weeks. Other phytocompounds (e.g. curcumin, quercetin) were evaluated in this study as well.
**Ginseng**	NCT00631852/Completed	Breast Cancer	A Phase II Biomarker Trial of Gelatin Encapsulated Extract of American Ginseng Root (LEAG) in Breast Cancer	The administration of American Ginseng Root extract was organised as follows: four 250 mg tablets daily for 5–14 days prior to surgery.
	NCT02603016/Completed	Lung Neoplasm Breast Carcinoma	Phase 1 Study of Clinical Nutrition That Research Safty and Efficacy in Lung Neoplasms And Breast Carcinoma	Two tablets of Ginseng were administered by mouth, twice a day for 42 days.
